# Supplementary material for: Everolimus Acts in Synergy with Vinorelbine to Suppress the Growth of Hepatocellular Carcinoma
Source: Int J Mol Sci. 2023 Dec 19;25(1):17. doi: 10.3390/ijms25010017 (PMC10779360; doi:10.3390/ijms25010017)
Supplement: Supplementary file 1 [file ijms-25-00017-s001.zip › ijms-2747018-supplementary.pdf]

__Supplementary File__

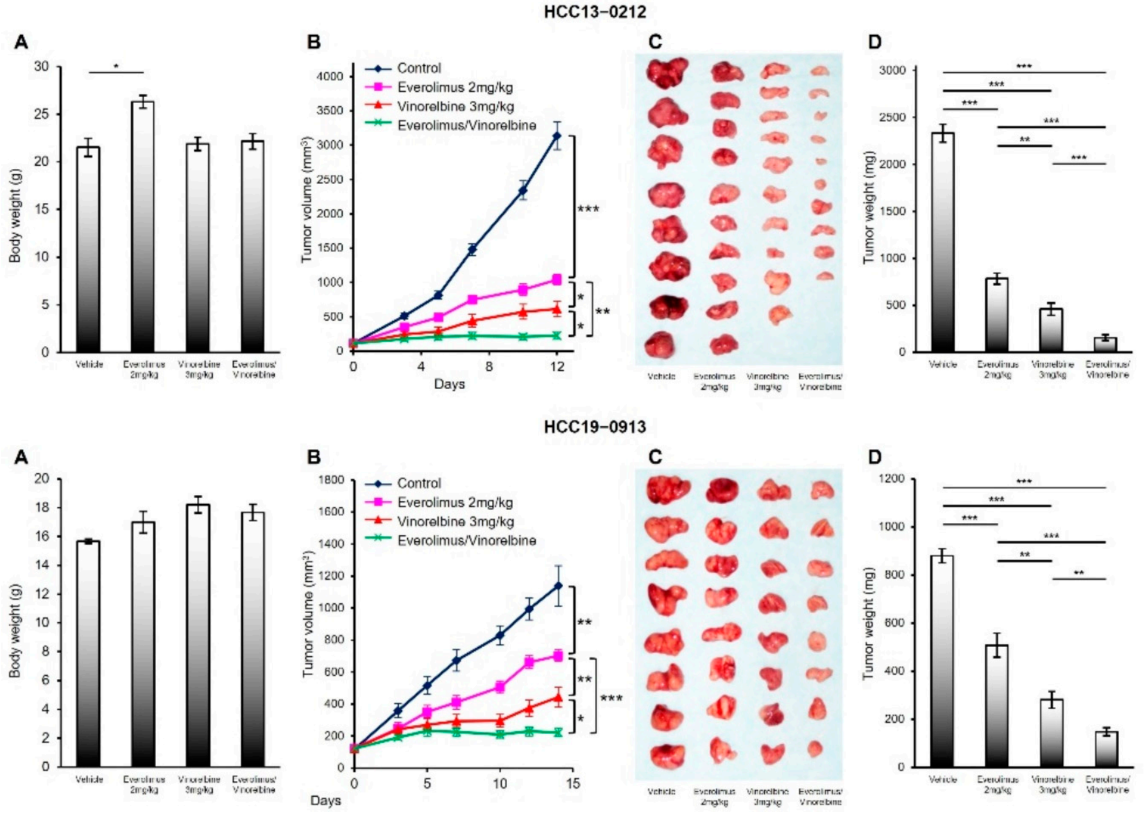

Supplementary Figure S1. Effects of everolimus, vinorelbine, and everolimus/vinorelbine on tumor growth in the HCC13−0212 and HCC19−0913 PDX models. HCC tumors were subcutaneously implanted into SCID mice, as described in the Materials and Methods section. Mice bearing the indicated tumors were randomly divided into four groups and orally treated with 200 μL of vehicle, 2 mg/kg everolimus once daily, 3 mg/kg vinorelbine once every 3.5 days, and 2 mg/kg everolimus plus 3 mg/kg vinorelbine on the indicated days. Each treatment arm comprised 8–10 independent tumor-bearing mice. (**A**) The mean of the body weight ± SEs at sacrifice, (**B**) the mean of the tumor volume ± SEs at given time points, (**C**) representative vehicle-, everolimus-, vinorelbine-, and everolimus-/vinorelbine-treated tumors, and (**D**) the mean of the corresponding tumor weight ± SEs are shown. Different asterisks (*) indicate significant differences (* $p < 0.05$; ** $p < 0.01$; *** $p < 0.001$; ANOVA followed by Tukey's test).

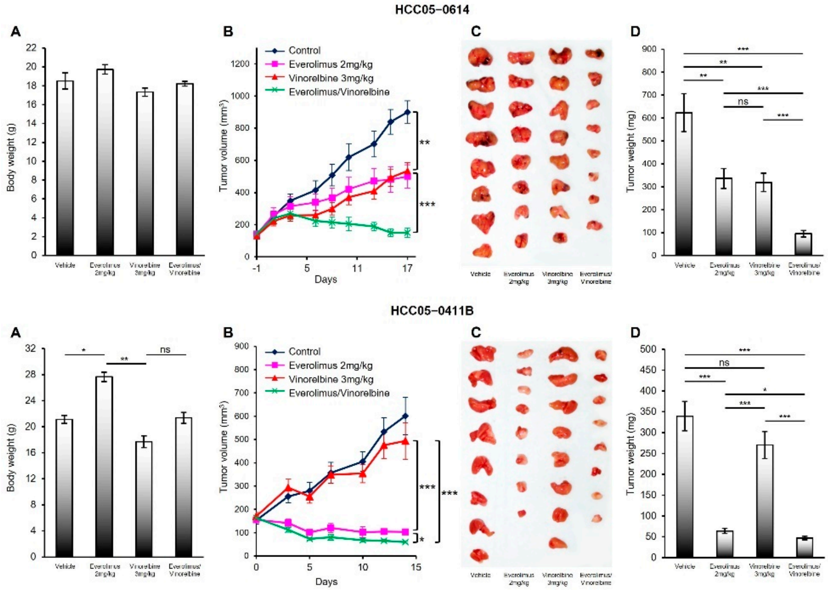

Supplementary Figure S2. Effects of everolimus, vinorelbine, and everolimus/vinorelbine on tumor growth in the HCC05−0614 and HCC05−411B PDX models. HCC tumors were subcutaneously implanted into SCID mice, as described in the Materials and Methods section. Mice bearing the indicated tumors were randomly divided into four groups and orally treated with 200 μL of vehicle, 2 mg/kg everolimus once daily, 3 mg/kg vinorelbine once every 3.5 days, and 2 mg/kg everolimus plus 3 mg/kg vinorelbine on the indicated days. Each treatment arm comprised 8–10 independent tumor-bearing mice. (**A**) The mean of the body weight ± SEs at sacrifice, (**B**) the mean of the tumor volume ± SEs at given time points, (**C**) representative vehicle-, everolimus-, vinorelbine-, and everolimus-/vinorelbine-treated tumors, and (**D**) the mean of the corresponding tumor weight ± SEs are shown. Different asterisks (*) indicate significant differences (* $p < 0.05$; ** $p < 0.01$; *** $p < 0.001$; ns, no significance; ANOVA followed by Tukey's test).

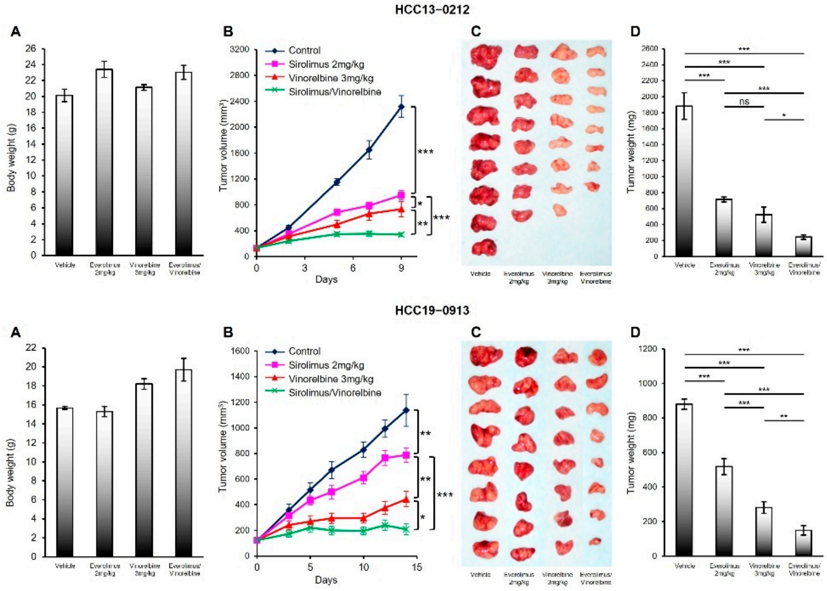

Supplementary Figure S3. Effects of sirolimus, vinorelbine, and sirolimus/vinorelbine on tumor growth in the HCC13−0212 and HCC19−0913 PDX models. HCC tumors were subcutaneously implanted into SCID mice, as described in the Materials and Methods section. Mice bearing the indicated tumors were randomly divided into four groups and orally treated with 200 μL of vehicle, 2 mg/kg sirolimus once daily, 3 mg/kg vinorelbine once every 3.5

days, and 2 mg/kg sirolimus plus 3 mg/kg vinorelbine on the indicated days. Each treatment arm comprised 8–10 independent tumor-bearing mice. (**A**) The mean of the body weight ± SEs at sacrifice, (**B**) the mean of the tumor volume ± SEs at given time points, (**C**) representative vehicle-, sirolimus-, vinorelbine-, and sirolimus-/vinorelbine-treated tumors, and (**D**) the mean of the corresponding tumor weight ± SEs are shown. Different asterisks (*) indicate significant differences (* $p < 0.05$; ** $p < 0.01$; *** $p < 0.001$; ns, no significance; ANOVA followed by Tukey's test).

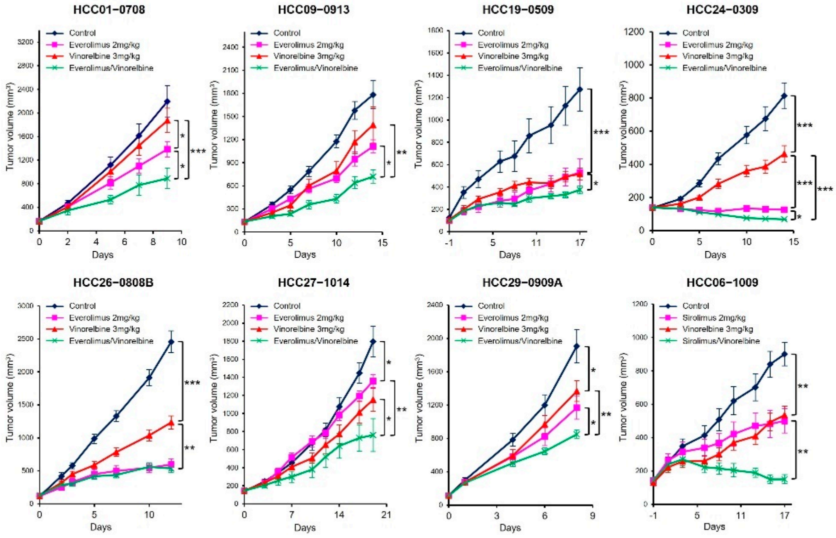

Supplementary Figure S4. Effects of everolimus (or sirolimus), vinorelbine, and everolimus (or sirolimus)/vinorelbine on tumor growth in the HCC PDX models. HCC tumors were subcutaneously implanted into SCID mice, as described in the Materials and Methods section. Mice bearing the indicated tumors were randomly divided into four groups and orally treated with 200 μL of vehicle, 2 mg/kg everolimus (or sirolimus) once daily, 3 mg/kg vinorelbine once every 3.5 days, and 2 mg/kg everolimus (or sirolimus) plus 3 mg/kg vinorelbine on the indicated days. Each treatment arm comprised 8–10 independent tumor-bearing mice. The mean of the tumor volume ± SEs at given time points are plotted. Different asterisks (*) indicate significant differences (* $p < 0.05$; ** $p < 0.01$; *** $p < 0.001$; ANOVA followed by Tukey's test).

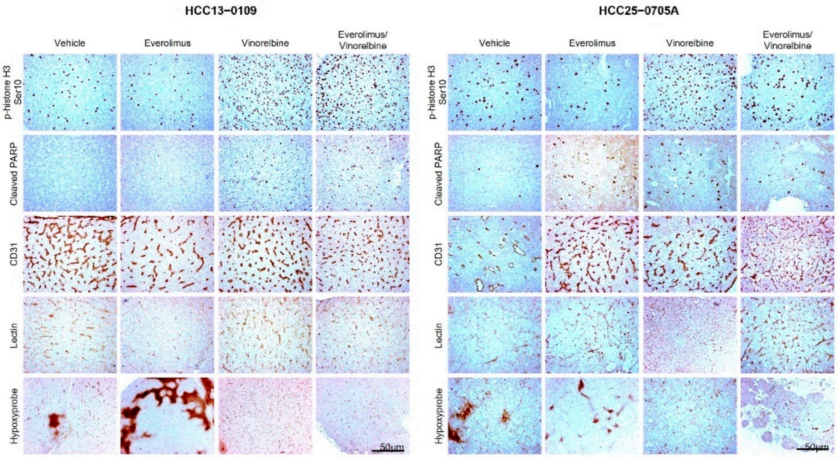

Supplementary Figure S5. Effects of everolimus, vinorelbine, and everolimus/vinorelbine on cell proliferation, apoptosis, blood vessel density, blood vessel normalization, and tumor hypoxia in the HCC13−0109 and HCC25−0705A PDX models. HCC tumors were subcutaneously implanted into SCID mice, as described in the Materials and Methods section. Mice bearing the indicated tumors were randomly divided into four groups and orally treated with 200 μL of

vehicle, 2 mg/kg everolimus once daily, 3 mg/kg vinorelbine once every 3.5 days, and 2 mg/kg everolimus plus 3 mg/kg vinorelbine for the indicated days. Each treatment arm comprised 8–10 independent tumor-bearing mice. Tumors collected 2 h after the last treatments were processed for IHC, as described in the Materials and Methods section. Representative images of tumor sections from vehicle- and drug-treated mice stained for p-histone H3 Ser10, cleaved PARP, CD31 (blood vessels), lectin, and Hypoxyprobe antibodies are shown. Images were captured using an Olympus BX60 microscope (Olympus, Japan). Scale bars: 50 μM.

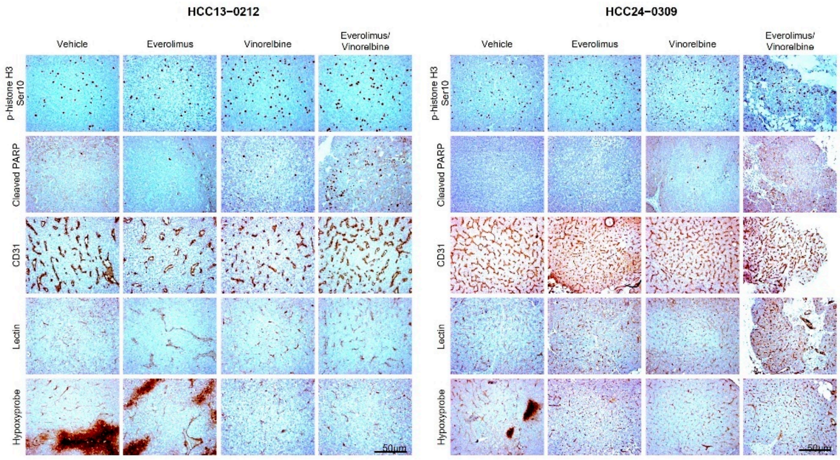

Supplementary Figure S6. Effects of everolimus, vinorelbine, and everolimus/vinorelbine on cell proliferation, apoptosis, blood vessel density, blood vessel normalization, and tumor hypoxia in the HCC13−0212 and HCC24−0309 PDX models. HCC tumors were subcutaneously implanted into SCID mice, as described in the Materials and Methods section. Mice bearing the indicated tumors were randomly divided into four groups and orally treated with 200 μL of vehicle, 2 mg/kg everolimus once daily, 3 mg/kg vinorelbine once every 3.5 days, and 2 mg/kg everolimus plus 3 mg/kg vinorelbine for the indicated days. Each treatment arm comprised 8–10 independent tumor-bearing mice. Tumors collected 2 h after the last treatments were processed for IHC, as described in the Materials and Methods section. Representative images of tumor sections from vehicle- and drug-treated mice stained for p-histone H3 Ser10, cleaved PARP, CD31 (blood vessels), lectin, and Hypoxyprobe antibodies are shown. Images were captured using an Olympus BX60 microscope (Olympus, Japan). Scale bars: 50 μM.

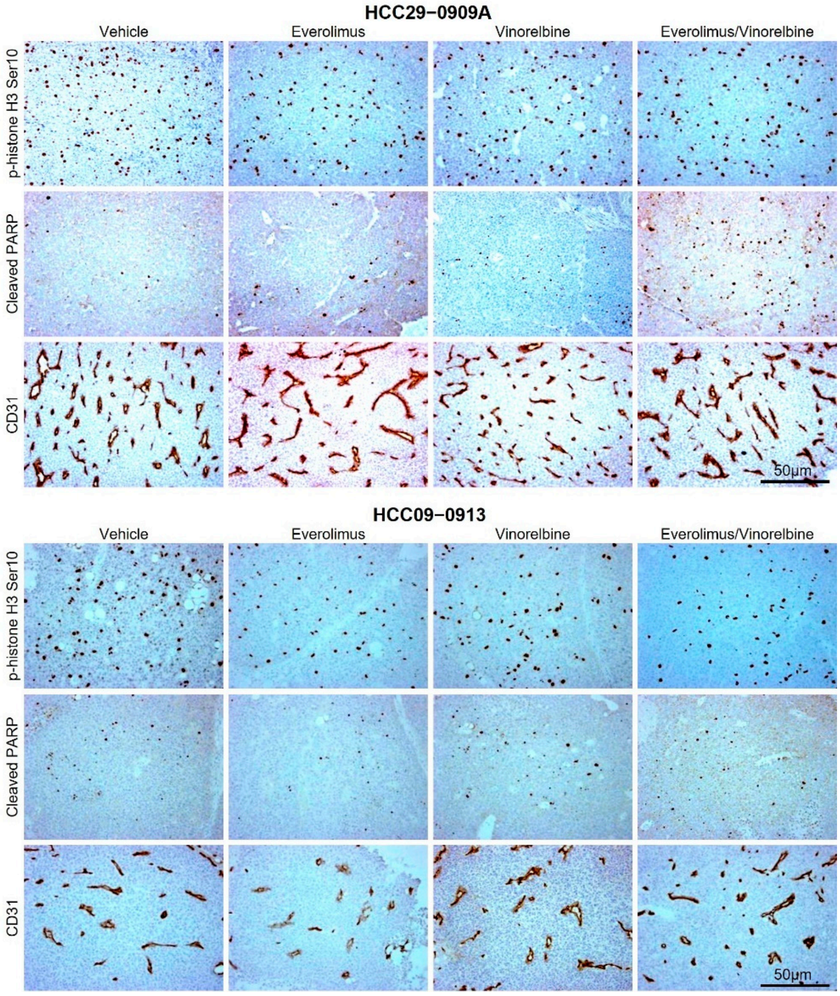

Supplementary Figure S7. Effects of everolimus, vinorelbine, and everolimus/vinorelbine on cell proliferation, apoptosis, and blood vessel density in the HCC29−0909A and HCC09−0913 PDX models. HCC tumors were subcutaneously implanted into SCID mice, as described in the Materials and Methods section. Mice bearing the indicated tumors were randomly divided into four groups and orally treated with 200 μL of vehicle, 2 mg/kg everolimus once daily, 3 mg/kg vinorelbine once every 3.5 days, and 2 mg/kg everolimus plus 3 mg/kg vinorelbine for the indicated days. Each treatment arm comprised 8–10 independent tumor-bearing mice. Tumors collected 2 h after the last treatments were processed for IHC, as described in the Materials and Methods section. Representative images of tumor sections from vehicle- and drug-treated mice stained for p-histone H3 Ser10, cleaved PARP, and CD31 (blood vessels) antibodies are shown. Images were captured using an Olympus BX60 microscope (Olympus, Japan). Scale bars: 50 μM.

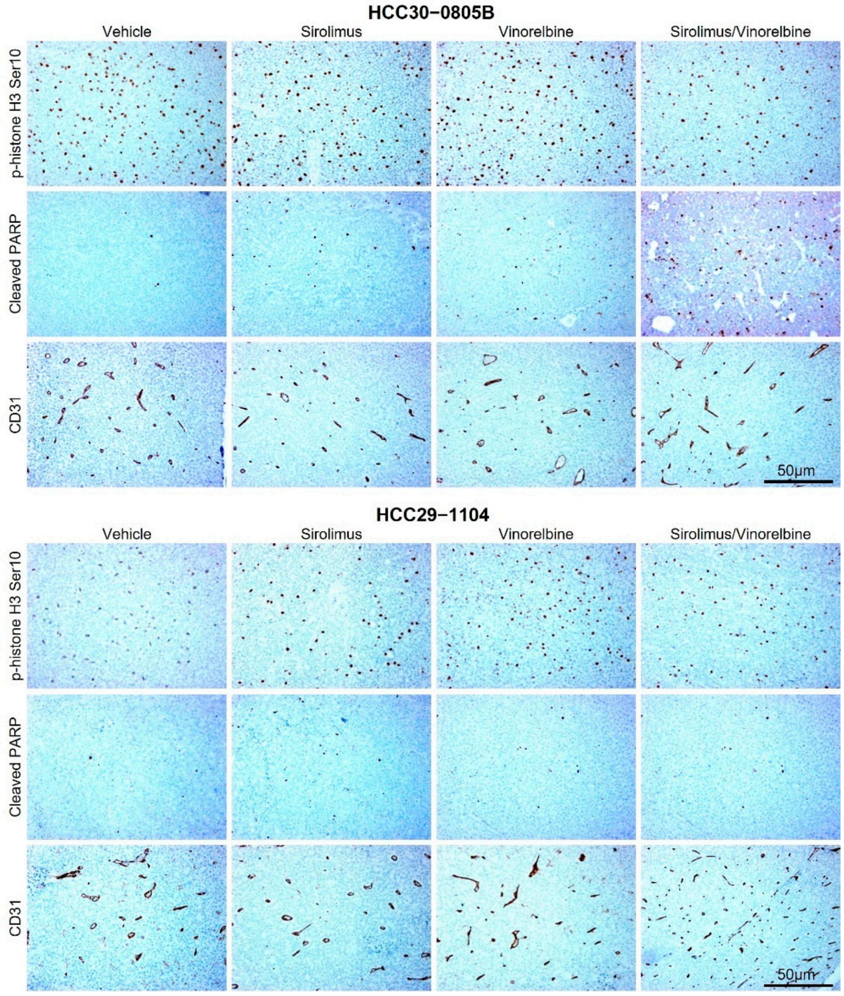

Supplementary Figure S8. Effects of sirolimus, vinorelbine, and sirolimus/vinorelbine on cell proliferation, apoptosis, and blood vessel density in the HCC30−0805B and HCC29−1104 PDX models. HCC tumors were subcutaneously implanted into SCID mice, as described in the Materials and Methods section. Mice bearing the indicated tumors were randomly divided into four groups and orally treated with 200 μL of vehicle, 2 mg/kg sirolimus once daily, 3 mg/kg vinorelbine once every 3.5 days, and 2 mg/kg sirolimus plus 3 mg/kg vinorelbine for the indicated days. Each treatment arm comprised 8–10 independent tumor-bearing mice. Tumors collected 2 h after the last treatments were processed for IHC, as described in the Materials and Methods section. Representative images of tumor sections from vehicle- and drug-treated mice stained for p-histone H3 Ser10, cleaved PARP, and CD31 (blood vessels) antibodies are shown. Images were captured using an Olympus BX60 microscope (Olympus, Japan). Scale bars: 50 μM.

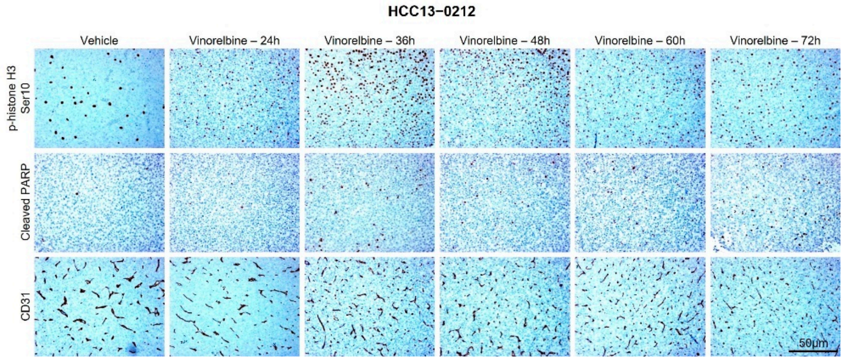

Supplementary Figure S9. Time-dependent effects of vinorelbine on cell proliferation, apoptosis, and blood vessel density in the HCC13−0212 PDX model. HCC13−0212 tumors were subcutaneously implanted into SCID mice, as described in the Materials and Methods section. Treatment started when the tumor sizes reached approximately 250–300 mm^3. Mice were intraperitoneally treated with vehicle (PBS) or 3 mg/kg vinorelbine. Tumors were harvested at specified intervals after treatment, including 0, 24, 36, 48, 60, and 72 h, and processed for IHC, as described in the Materials and Methods section. Representative images of tumor sections from vehicle- and drug-treated mice stained for p-histone H3 Ser10, cleaved PARP, and CD31 (blood vessels) antibodies are shown. Images were captured using an Olympus BX60 microscope (Olympus, Japan). Scale bars: 50 μM.

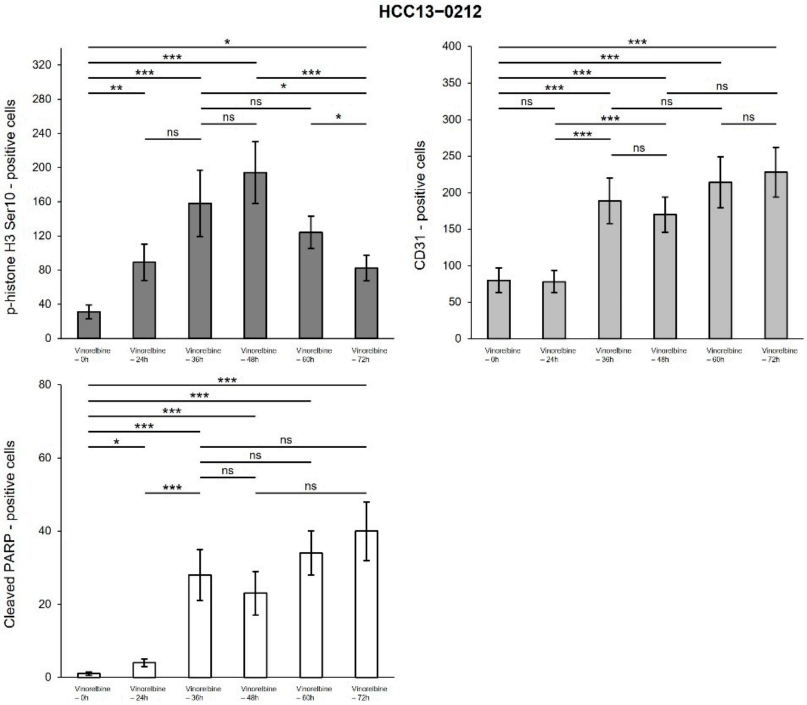

Supplementary Figure S10. Quantification analysis of vinorelbine-induced cell proliferation, apoptosis, and blood vessel density in the HCC13−0212 PDX model. Mice bearing the HCC13−0212 tumors were intraperitoneally treated with vehicle (PBS) or 3 mg/kg vinorelbine. Tumors were harvested at specified intervals after treatment, including 0, 24, 36, 48, 60, and 72 h, and processed for IHC, as described in the Materials and Methods section. Tumor sections from vehicle- and drug-treated mice were immunostained with p-histone H3 Ser10, cleaved PARP, and CD31 (blood vessels) antibodies to assess cell proliferation, apoptosis, and total blood vessels, respectively. At least 10 fields at a magnification of 100x were randomly captured on each IHC-stained slide using an Olympus BX60 microscope (Olympus, Japan). The total number of p-histone H3 Ser10-, cleaved PARP-, and CD31-positive cells per captured field

were counted. The mean of the positive cells ± SEs are plotted, and differences between groups were compared (* $p < 0.05$; ** $p < 0.01$; *** $p < 0.001$; ns, no significance; ANOVA followed by Tukey's test).

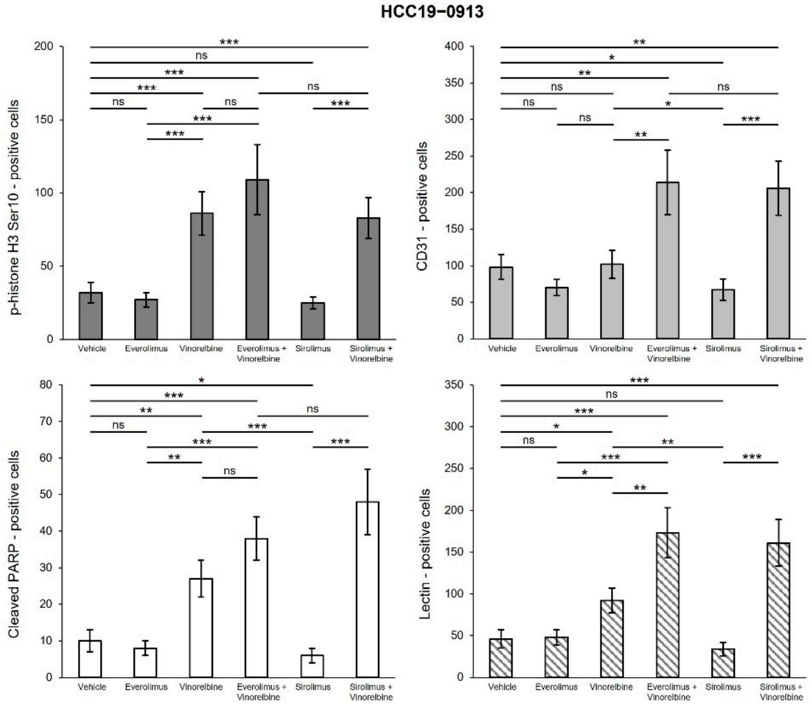

Supplementary Figure S11. Quantification analysis of everolimus-, sirolimus-, vinorelbine-, everolimus-/vinorelbine-, and sirolimus-/vinorelbine-induced cell proliferation, apoptosis, blood vessel density, and blood vessel normalization in the HCC19−0913 PDX model. HCC19−0913 tumors were subcutaneously implanted into SCID mice, as described in the Materials and Methods section. Mice bearing the indicated tumors were randomly divided into six groups and orally treated with 200 μL of vehicle, 2 mg/kg everolimus once daily, 3 mg/kg vinorelbine once every 3.5 days, 2 mg/kg everolimus plus 3 mg/kg vinorelbine, 2 mg/kg sirolimus once daily, and 2 mg/kg sirolimus plus 3 mg/kg vinorelbine for the indicated days. Each treatment arm comprised 8–10 independent tumor-bearing mice. Tumors collected 2 h after the last treatments were processed for IHC, as described in the Materials and Methods section. Tumor sections from the vehicle- and drug-treated mice were immunostained with p-histone H3 Ser10, cleaved PARP, CD31 (blood vessels), and lectin antibodies to assess cell proliferation, apoptosis, total blood vessels, and blood vessel normalization, respectively. At least 10 fields at a magnification of 100x were randomly captured on each IHC-stained slide using an Olympus BX60 microscope (Olympus, Japan). The total number of p-histone H3 Ser10-, cleaved PARP-, CD31-, and lectin-positive cells per captured field were counted. The mean of the positive cells ± SEs are plotted, and differences between groups were compared (* $p < 0.05$; ** $p < 0.01$; *** $p < 0.001$; ns, no significance; ANOVA followed by Tukey's test).

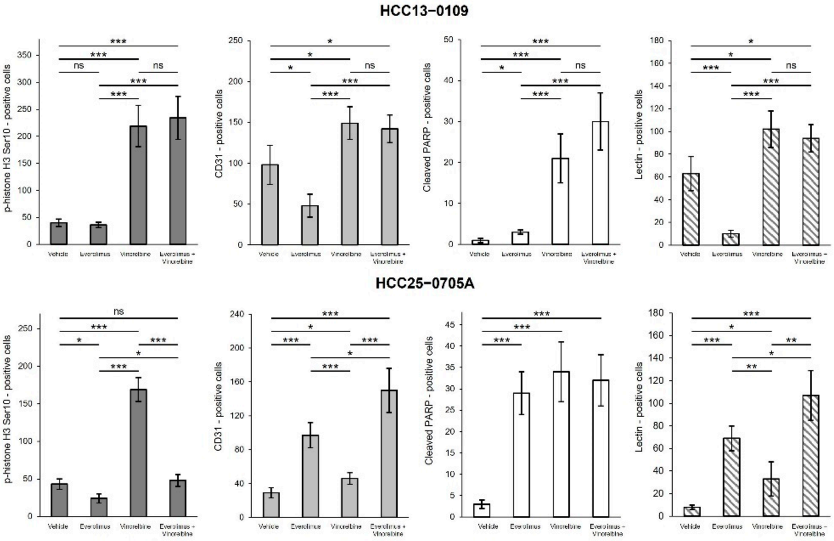

Supplementary Figure S12. Quantification analysis of everolimus-, vinorelbine-, and everolimus-/vinorelbine-induced cell proliferation, apoptosis, blood vessel density, and blood vessel normalization in the HCC13−0109 and HCC25−0705A PDX models. HCC tumors were subcutaneously implanted into SCID mice, as described in the Materials and Methods section. Mice bearing the indicated tumors were randomly divided into four groups and orally treated with 200 μL of vehicle, 2 mg/kg everolimus once daily, 3 mg/kg vinorelbine once every 3.5 days, and 2 mg/kg everolimus plus 3 mg/kg vinorelbine for the indicated days. Each treatment arm comprised 8–10 independent tumor-bearing mice. Tumors collected 2 h after the last treatments were processed for IHC, as described in the Materials and Methods section. Tumor sections from the vehicle- and drug-treated mice were immunostained with p-histone H3 Ser10, cleaved PARP, CD31 (blood vessels), and lectin antibodies to assess cell proliferation, apoptosis, total blood vessels, and blood vessel normalization, respectively. At least 10 fields at a magnification of 100x were randomly captured on each IHC-stained slide using an Olympus BX60 microscope (Olympus, Japan). The total number of p-histone H3 Ser10-, cleaved PARP-, CD31-, and lectin-positive cells per captured field were counted. The mean of the positive cells ± SEs are plotted, and differences between groups were compared (* $p < 0.05$; ** $p < 0.01$; *** $p < 0.001$; ns, no significance; ANOVA followed by Tukey's test).

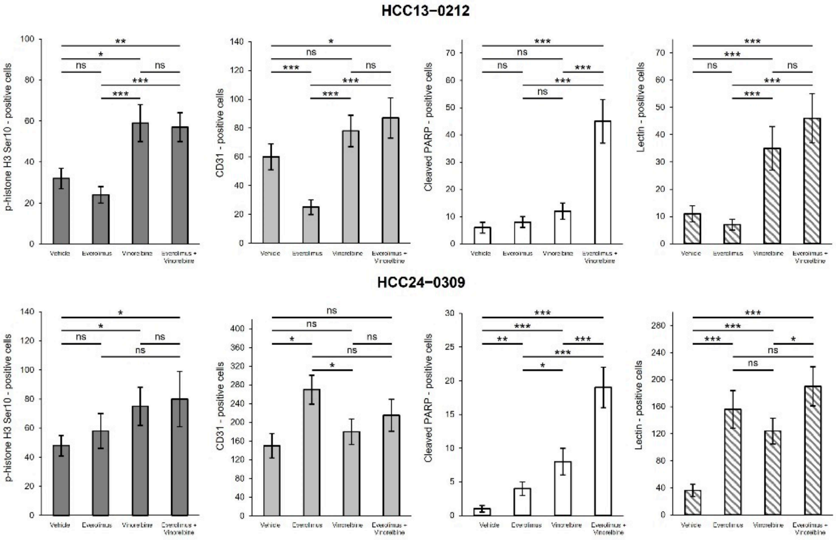

Supplementary Figure S13. Quantification analysis of everolimus-, vinorelbine-, and everolimus-/vinorelbine-induced cell proliferation, apoptosis, blood vessel density, and blood vessel normalization in the HCC13−0212 and HCC24−0309

PDX models. HCC tumors were subcutaneously implanted into SCID mice, as described in the Materials and Methods section. Mice bearing the indicated tumors were randomly divided into four groups and orally treated with 200 μL of vehicle, 2 mg/kg everolimus once daily, 3 mg/kg vinorelbine once every 3.5 days, and 2 mg/kg everolimus plus 3 mg/kg vinorelbine for the indicated days. Each treatment arm comprised 8–10 independent tumor-bearing mice. Tumors collected 2 h after the last treatments were processed for IHC, as described in the Materials and Methods section. Tumor sections from the vehicle- and drug-treated mice were immunostained with p-histone H3 Ser10, cleaved PARP, CD31 (blood vessels), and lectin antibodies to assess cell proliferation, apoptosis, total blood vessels, and blood vessel normalization, respectively. At least 10 fields at a magnification of 100x were randomly captured on each IHC-stained slide using an Olympus BX60 microscope (Olympus, Japan). The total number of p-histone H3 Ser10-, cleaved PARP-, CD31-, and lectin-positive cells per captured field were counted. The mean of the positive cells ± SEs are plotted, and differences between groups were compared (* $p < 0.05$; ** $p < 0.01$; *** $p < 0.001$; ns, no significance; ANOVA followed by Tukey's test).

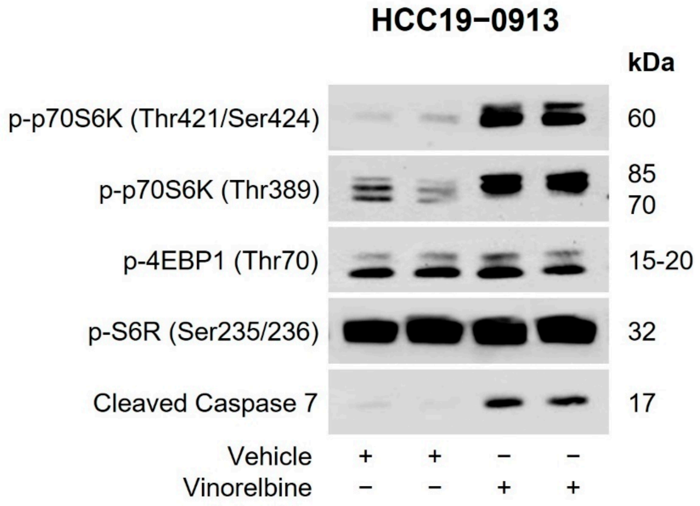

Supplementary Figure S14. Effects of vinorelbine on AKT/p70S6K/4EBP1, ERK1/2, and cell cycle regulators in the HCC19−0913 PDX model. HCC19−0913 tumors were subcutaneously implanted into SCID mice, as described in the Materials and Methods section. Mice bearing the indicated tumors mice were treated with 200 μL of vehicle or 3 mg/kg vinorelbine once. Each treatment arm comprised 2 independent tumor-bearing mice. Tumors were collected after 3 days and subjected to a Western blot analysis, as described in the Materials and Methods section. Representative blots incubated with the indicated antibodies and molecular markers of the proteins (kDa) are shown.

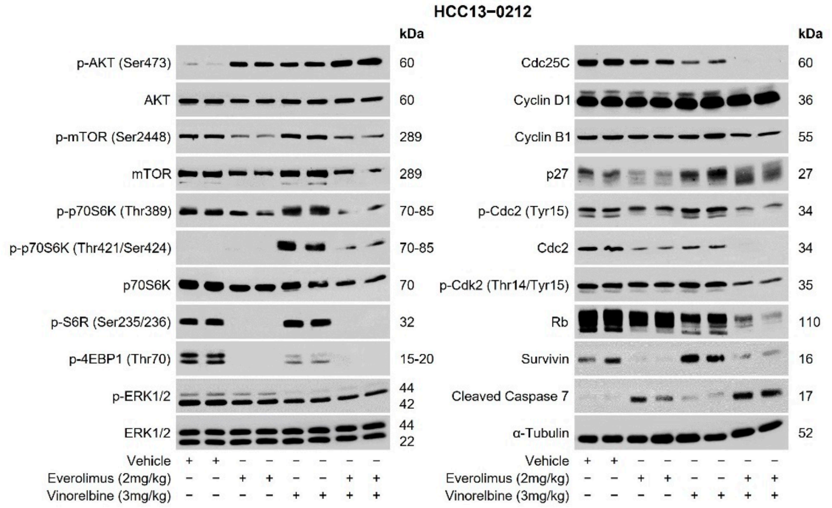

Supplementary Figure S15. Effects of everolimus, vinorelbine, and everolimus/vinorelbine on AKT/p70S6K/4EBP1, ERK1/2, and cell cycle regulators in the HCC13−0212 PDX model. HCC13−0212 tumors were subcutaneously implanted into SCID mice, as described in the Materials and Methods section. Mice bearing the indicated tumors were randomly divided into four groups and orally treated with 200 μL of vehicle, 2 mg/kg everolimus once daily, 3 mg/kg vinorelbine once every 3.5 days, and 2 mg/kg everolimus plus 3 mg/kg vinorelbine for the indicated days. Each treatment arm comprised 8–10 independent tumor-bearing mice. Tumors were collected and subjected to a Western blot analysis, as described in the Materials and Methods section. Representative blots incubated with the indicated antibodies and molecular markers of the proteins are shown.

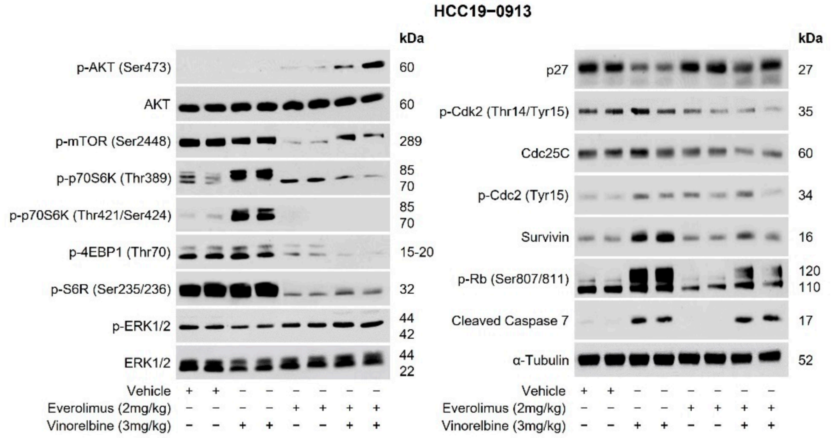

Supplementary Figure S16. Effects of everolimus, vinorelbine, and everolimus/vinorelbine on AKT/p70S6K/4EBP1, ERK1/2, and cell cycle regulators in the HCC19−0913 PDX model. HCC19−0913 tumors were subcutaneously implanted into SCID mice, as described in the Materials and Methods section. Mice bearing the indicated tumors were randomly divided into four groups and orally treated with 200 μL of vehicle, 2 mg/kg everolimus once daily, 3 mg/kg vinorelbine once every 3.5 days, and 2 mg/kg everolimus plus 3 mg/kg vinorelbine for the indicated days. Each treatment arm comprised 8–10 independent tumor-bearing mice. Tumors were collected and subjected to a Western blot analysis, as described in the Materials and Methods section. Representative blots incubated with the indicated antibodies and molecular markers of the proteins are shown.

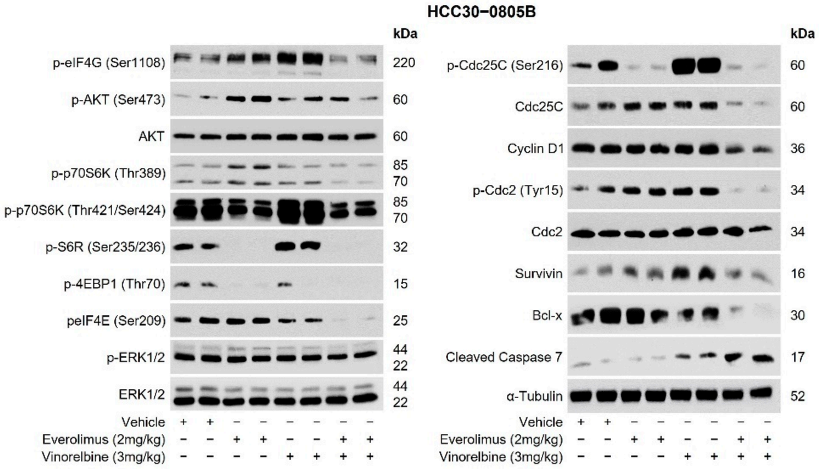

Supplementary Figure S17. Effects of sirolimus, vinorelbine, and sirolimus/vinorelbine on AKT/p70S6K/4EBP1, ERK1/2, and cell cycle regulators in the HCC30−0805B PDX model. HCC30−0805B tumors were subcutaneously implanted into SCID mice, as described in the Materials and Methods section. Tumor-bearing mice were randomly divided into four groups and orally treated with 200 µL of vehicle, 2 mg/kg sirolimus once daily, 3 mg/kg vinorelbine once every 3.5 days, and 2 mg/kg sirolimus plus 3 mg/kg vinorelbine for the indicated days. Each treatment arm comprised 8–10 independent tumor-bearing mice. Tumors were collected and subjected to a Western blot analysis, as described in the Materials and Methods section. Representative blots incubated with the indicated antibodies and molecular markers of the proteins are shown.

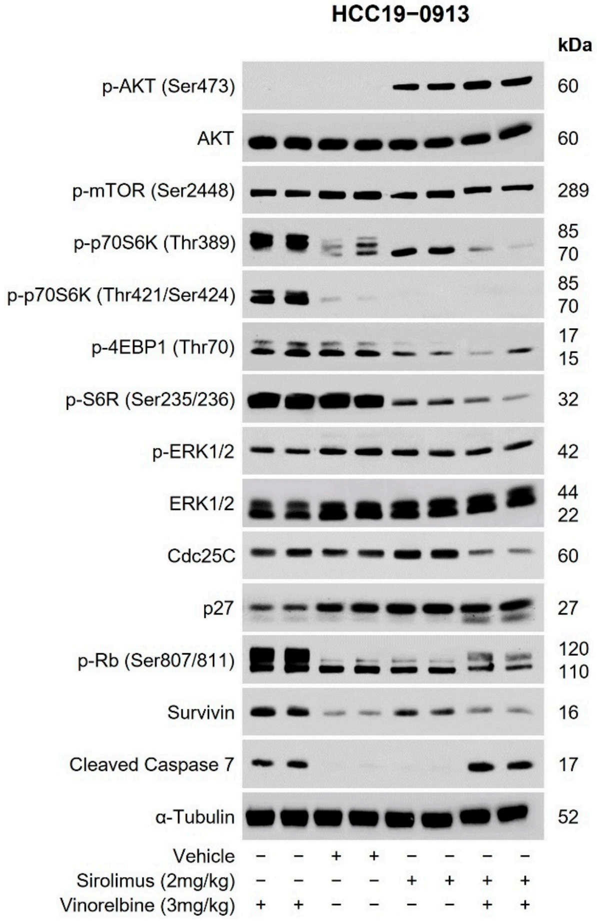

Supplementary Figure S18. Effects of sirolimus, vinorelbine, and sirolimus/vinorelbine on AKT/p70S6K/4EBP1, ERK1/2, and cell cycle regulators in the HCC19−0913 PDX model. HCC19−0913 tumors were subcutaneously implanted into SCID mice, as described in the Materials and Methods section. Mice bearing the indicated tumors were randomly divided into four groups and orally treated with 200 μL of vehicle, 2 mg/kg sirolimus once daily, 3 mg/kg vinorelbine once every 3.5 days, and 2 mg/kg sirolimus plus 3 mg/kg vinorelbine for the indicated days. Each treatment arm comprised 8–10 independent tumor-bearing mice. Tumors were collected and subjected to a Western blot analysis, as described in the Materials and Methods section. Representative blots incubated with the indicated antibodies and molecular markers of the proteins are shown.
